# Enhancing the Chloride Adsorption and Durability of Sulfate-Resistant Cement-Based Materials by Controlling the Calcination Temperature of CaFeAl-LDO

**DOI:** 10.3390/ma18214884

**Published:** 2025-10-24

**Authors:** Lei Yang, Xin Zhao, Shaonan Cai, Minqi Hua, Jijiang Liu, Hui Liu, Junyi Wu, Liming Pang, Xinyu Gui

**Affiliations:** 1 School of Urban Construction, Changzhou University, Changzhou 213164, China; 2 School of Civil Engineering & Architecture, Wuhan University of Technology, Wuhan 430070, China; 3 Jinzhou Engineering Construction Command Headquarters, China Railway Shenyang Group Co., Ltd., Jinzhou 121000, China

**Keywords:** marine concrete, layered double oxide, chloride adsorption, concrete durability, sulphate-resistant Portland cement

## Abstract

Chloride-ion (Cl^−^)-induced corrosion of steel bars is a major threat to the durability of marine concrete structures. To address this, a type of calcined CaFeAl-layered double oxide (LDO-CFA) with different calcination temperatures was used to enhanced the Cl^−^ adsorption, compressive strength, and corrosion resistance of sulphate-resistant Portland cement (SRPC)-based materials. Experimental results demonstrated that LDO-CFA exhibited high Cl^−^ adsorption capacity in both CPSs and cement-based materials. Specifically, LDO-750-CFA reached 1.98 mmol/g in CPSs—60.1% higher than LDHs-CFA—and followed the Langmuir model, indicating monolayer adsorption. It also reduced the free Cl^−^ content of SRPC paste to 0.255–0.293% after 28 days, confirming its sustained adsorption over extended curing. Furthermore, LDO-CFA positively influenced the compressive strength at all curing ages. At an optimal dosage of 0.8 wt.%, LDO-750-CFA paste significantly improved the compressive strength, increasing it by 22.1% at 7 days and 15.6% at 28 days compared to the control. Electrochemical analysis confirmed the superior corrosion resistance of the LDO-750-CFA system. The property enhancement originated from LDO-750-CFA’s synergistic effects, which included pore refinement, increased tortuosity, Cl^−^ adsorption by structural memory, a PVP-induced passive film, and PVP-improved dispersion. Overall, this work provides a framework for developing LDO-750-CFA-based composites, paving the way for more durable marine concrete.

## 1. Introduction

As a fundamental material in marine engineering, marine concrete has emerged as the predominant choice for coastal infrastructure owing to its exceptional anti-corrosion and penetration resistances in harsh marine environments [1,2,3]. However, chloride-ion (Cl^−^)-induced steel bar corrosion remains a critical factor in the degradation of marine concrete. Research demonstrated that Cl^−^ at high concentrations in seawater infiltrated concrete through multiple transport mechanisms, including capillary absorption, concentration gradient-driven diffusion, and electrochemical migration [4]. Critical Cl^−^ accumulation at the steel–concrete interface breaks down the passive film, inducing steel corrosion through macrocell corrosion and pitting [5,6]. Therefore, enhancing the Cl^−^ adsorption is critical for ensuring structural longevity and durability.

By reducing permeability through advanced material formulations and protective treatments, concrete structures can resist environmental degradation, minimize their maintenance costs, and achieve service lives exceeding 50–100 years. The service life of concrete in complex environments can be significantly extended by controlling Cl^−^ diffusion and migration, as evidenced by studies [7,8]. Specifically, to improve marine concrete performance, two primary strategies are recommended, both contributing to superior corrosion resistance: (1) enhancement of Cl^−^ adsorption behavior in cement-based material, and (2) microstructural compactness of marine concrete [9,10]. For instance, Wang et al. [11] demonstrated that aluminum oxide exhibits superior Cl^−^ adsorption capacity compared to aluminum hydroxide in cementitious systems. Both compounds enhanced Cl^−^ binding by promoting Friedel’s salt (FS) formation throughout the curing process. Xiang et al. [12] found that incorporating nano-silica dioxide improved the durability of concrete by densifying its microstructure. However, conventional compaction methods have reached their limits, making it increasingly difficult to further enhance concrete density. This is because the hydration and hardening of concrete involve a dynamic physic-chemical equilibrium, which does not fully capture the evolution of the pore structure after hydration. Therefore, the synergistic integration of Cl^−^ binding and micro-nano pore-filling technologies has emerged as a pivotal research focus in enhancing concrete durability.

Layered double hydroxides (LDHs) are a class of layered structured two-dimensional nanosheet materials, and their chemical composition is depicted as [M^2+^_1−x_M^3+^_x_(OH)_2_](A^n−^)_x/n_·yH_2_O [13,14]. Owing to the relatively weak hydrogen bonding of interlayer anions, LDHs allow for anion exchange via strong electrostatic interactions, making them effective adsorbents for Cl^−^ removal from solution [15]. The CaLiAl-NO_3_ LDHs synthesized by He et al. [16] served as an effective Cl^−^ adsorbent (41.47 mg/g) and a hydration promoter, significantly enhancing the Cl^−^ binding capacity of the cement sample with 1.5% CaLiAl-NO_3_ LDHs by 42.83% at 28 d. Xu et al. [17] demonstrated that while all four CaAl/Fe-NO_3_/NO_2_ LDHs adsorb Cl^−^ in the concrete pore solution, their stability and capacity are governed by the binding energy between intercalated ions and the host layers, with a higher binding energy leading to greater resistance to anion exchange and thus a higher saturated Cl^−^ adsorption content. Jiang et al. [18] demonstrated the multiple synergistic corrosion inhibition effects in Cl^−^ environments by using inhibitor-modified Mg/Al-pAB-LDHs, which also concurrently enhanced the mortar’s mechanical properties and chloride resistance. In addition to their well-documented ion-exchange capabilities, LDHs exhibit a unique structural memory effect. Upon calcination (typically at 400–600 °C), LDHs transform into layered double oxide (LDO), which can reconstruct the original layered structure through rehydration in aqueous environments [19,20]. During this reconstruction, LDO adsorbed anions from the solution and showed a higher adsorption capacity than the original LDHs, due to the formation of high-surface-area metastable phases after calcination [19]. Gao et al. [20] stated that MgAl-LDO demonstrated a high Cl^−^ adsorption capacity of 31 mg/g in recycled aggregate leachate. Additionally, the small size effect of LDO contributed to pore filling in concrete, which enhanced its compactness and improved the Cl^−^ penetration resistance [20,21]. However, studies [21,22] indicated that at elevated temperatures (typically above 600 °C), the reconstruction ability of LDHs was significantly reduced. As a result, the calcined product could no longer fully revert to the original LDHs’ layered structure in anionic solutions. Nevertheless, it retained a relatively high anion adsorption capacity compared to uncalcined LDHs [23]. Consequently, optimization is required to determine the ideal balance between the calcination temperature of LDO and its incorporation for Cl^−^ adsorption, ensuring maximum adsorption efficiency.

The strong charge density of LDHs promoted agglomeration, which reduced the Cl^−^ adsorption capacity and the compactness of cement-based materials. Thus, in order to improve the dispersion of micro-nano materials and strengthen the corrosion resistance of cement-based systems, it was essential to develop specialized dispersants for marine concrete applications [24]. Polyvinylpyrrolidone (PVP) has demonstrated effectiveness in improving the dispersion and reducing the size of suspended solid particles [25]. These characteristics position PVP as a promising dispersant for enhancing the uniformity and stability of nano materials. In addition, sulphate-resistant Portland cement (SRPC) contains a higher proportion of tetracalcium aluminoferrite (C_4_AF, 4CaO·Al_2_O_3_·Fe_2_O_3_). The Fe-phase hydrates rapidly, resulting in a denser matrix that offers improved microstructure compactness. The hydration products of the Fe-phase, such as (A, F)H_3_, possess a high specific surface area, which enhances the physical adsorption of corrosive ions and promotes the chemical binding of Cl^−^. As a result, SRPC has exhibited superior corrosion resistance compared to the other three major types of cement clinker. This proved to be particularly effective in enhancing concrete durability against corrosive and marine environments [26,27]. Therefore, in the harsh marine environment, because of its excellent resistance to seawater erosion, SRPC has a broad application prospect in marine dam projects, water conservancy facilities and undersea tunnels severely affected by severe sulfate erosion, as it resists chemical erosion and the high salinity environment.

Based on the aforementioned issues, the aim of this study was to calcine CaFeAl-LDO (LDO-CFA) at high temperatures (550–750 °C) and evaluate the Cl^−^ adsorption behavior in both concrete pore solutions (CPSs) and SPRC-based materials. Subsequently, the effects of LDO-CFA on the mechanical properties and corrosion resistance of the SRPC-based materials were systematically investigated. The micro-mechanisms of different phenomena occurring in the CPSs and SRPC-based materials containing Cl^−^ were characterized using X-ray diffraction (XRD), thermogravimetry–derivative thermogravimetry (TG-DTG), and pore structure analysis. Through a comprehensive analysis in both CPSs and actual SRPC-based environments, LDO-CFA demonstrates significant potential as an efficient corrosion resistance agent specifically for offshore cement structures.

## 2. Experimental

### 2.1. Materials

**Figure 1:**
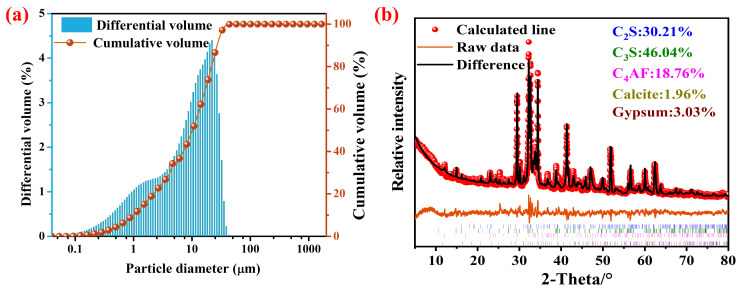
Particle size distribution (**a**) and XRD analysis of SRPC: (C=CaO, S=SiO_2_, A=Al_2_O_3_, F=Fe_2_O_3_) (**b**).

The cementitious material used in this experiment was SRPC, supplied by Shandong Qiyin Cement Co., Ltd. (Zibo, China). It has a density of 3.21 g/cm^3^ and a specific surface area of 388 m^2^/kg. The chemical composition and particle size distribution of the SRPC are provided in Table 1 and Figure 1a, respectively. The XRD patterns of the SRPC in Figure 1b demonstrated a cement phase composition dominated by 46.04% tricalcium silicate (C_3_S, 3CaO·SiO_2_) with undetectable tricalcium aluminate (C_3_A, 3CaO·Al_2_O_3_) content, fully compliant with the Chinese Standard GB/T 748-2023 [28] requirements (C_3_S ≤ 50.0%; C_3_A ≤ 3.0%). The sand employed in the experiments was standard sand, characterized by a fineness modulus of 2.87. The K29-32 PVP, a high-molecular-weight amphiphilic copolymer containing bifunctional groups, was supplied by Shanghai Macklin Biochemical Co., Ltd. (Shanghai, China). Carbon steel bars (Grade 45) with dimensions of φ6 mm × 100 mm were sourced from Xinkai Chuang Steel Company, Wuxi, China. The deionized water was prepared by the UPL-II-120H ultra-pure water machine (Sichuan Yupu Ultra-Pure Technology Co., Ltd., Chengdu, China).

### 2.2. Preparation of Calcined LDO-CFA

According to previous studies, CaFeAl-NO_3_ LDHs (LDHs-CFA) were synthesized by a co-precipitation method [29]. Then, using LDHs-CFA as the precursor, LDO-CFA was synthesized by calcination at 550 °C and 750 °C for 3 h with a heating rate of 5 °C/min. After calcination, the specimens were cooled naturally inside the furnace to room temperature, collected into sealed centrifuge tubes, and stored in a desiccator. The resulting calcined products were denoted as LDO-550-CFA and LDO-750-CFA, respectively.

### 2.3. Extraction Process of Concrete Pore Solutions

**Figure 2:**
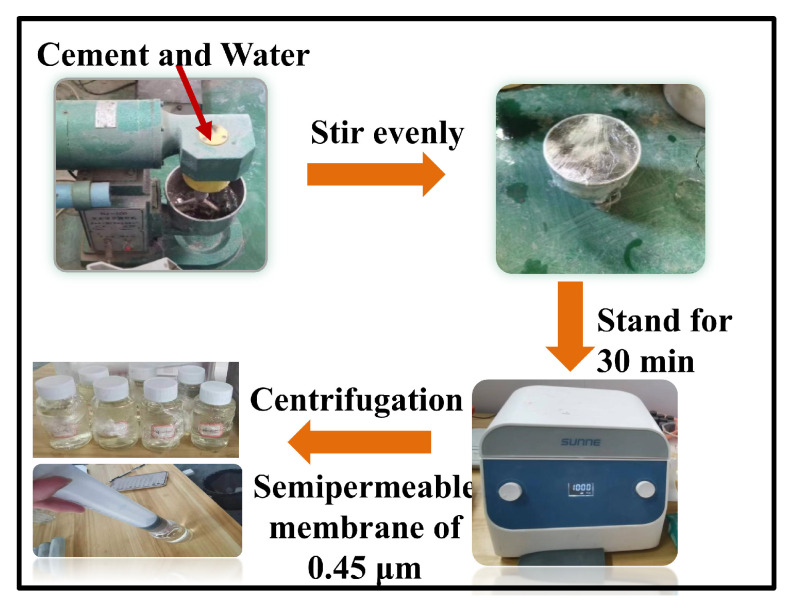
The specific extraction process of the CPSs.

To investigate the Cl^−^ adsorption behavior and competitive mechanisms of LDO-CFA in the presence of multiple anions, the CPSs were prepared to replicate the environment of cement-based materials. To prepare the CPSs, SRPC and water were mixed at a 1:1 ratio. The mixture was stirred and then stood for 30 min. Then, the supernatant was collected and filtered through a 0.45 μm membrane to obtain the final CPSs. The ion concentrations of the main component are shown in Table 2. The pH of the CPSs was 12.76, compared to approximately 12.5 in both saturated Ca(OH)_2_ and the simulated solutions [16,17,20]. Furthermore, as cement is a complex multi-component system, the ionic composition and concentration in its actual pore solution deviate markedly from those in the simplified simulated solution. Thus, a saturated Ca(OH)_2_ solution does not accurately represent the chemical environment during cement hydration. Using such simulated solutions to investigate Cl^−^ adsorption and phase evolution in LDO-CFA has limitations and may not reflect real conditions. In this experiment, the CPSs were directly employed to investigate the Cl^−^ adsorption by LDO-CFA. The specific extraction process of the CPSs is shown in Figure 2.

### 2.4. Preparation of Specimens

**Figure 3:**
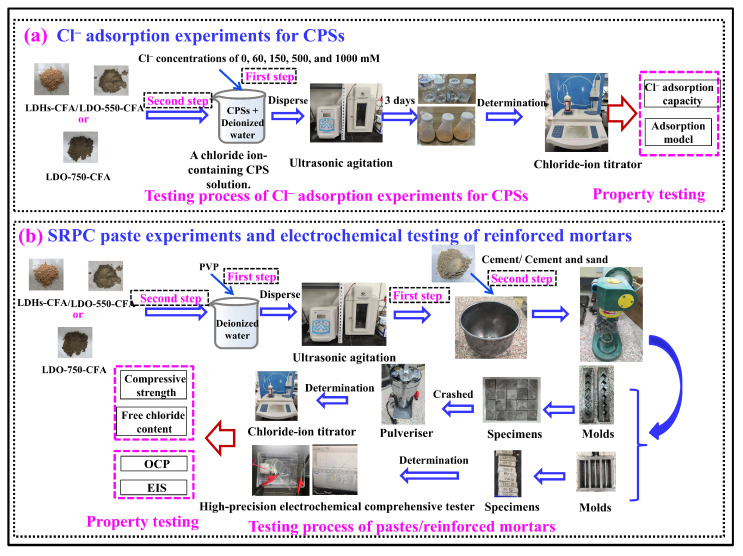
The procedure for specimen preparation and characterization: Cl^−^ adsorption experiments for CPSs (**a**); SRPC paste experiments and electrochemical testing of reinforced mortars (**b**).

Figure 3 illustrates the schematic of the procedural workflow, which includes the Cl^−^ adsorption experiments for CPSs, SRPC paste experiments, and electrochemical tests for the reinforced mortar. Among them, in the Cl^−^ adsorption experiments, different amounts of NaCl were added to 50 mL of the prepared CPSs to obtain Cl^−^ concentrations of 0, 60, 150, 500, and 1000 mM. Then, 1.0 g LDO-CFA was introduced into each solution. The mixtures were dispersed via ultrasonication for 2 min to form a stable suspension, purged with N_2_, sealed, and kept at 20 °C for 3 days to reach adsorption equilibrium. After 3 days of adsorption, residual Cl^−^ concentrations were measured using a Cl^−^ concentration tester. Additionally, the data were fitted to commonly used adsorption isotherm models—Langmuir, Freundlich, and Temkin—to analyze their adsorption mechanism. In the SRPC paste experiments, the contents of LDHs-CFA, LDO-550-CFA, and LDO-750-CFA were varied at 0.3, 0.8, and 1.5%. Cement slurry specimens were prepared with a water–cement ratio of 0.35, where NaCl (3.5% by mass of water) was added to simulate saline conditions. To assess durability in chloride-rich environments, the cast specimens (20 mm^3^ cubes) underwent 7 days and 28 days of moist curing before measuring the compressive strength and Cl^−^ adsorption performance. The mix proportions used for the electrochemical tests of the reinforced mortars are listed in Table 3. In the electrochemical testing of the reinforced mortars, PVP was initially dissolved in water and ultrasonically dispersed for 5 min to prevent particle agglomeration of LDO-CFA. The LDO-CFA was then added to this solution and further dispersed ultrasonically for 1 min. The resulting homogeneous dispersion was then added to the cementitious admixture and sand.

### 2.5. Test Method

#### 2.5.1. Chloride Ion Adsorption Determination

The Cl^−^ adsorption capacity of specimens was determined by potentiometric titration. For the CPSs, the Cl^−^ concentration was determined by a Cl^−^ concentration tester equipped with a ZDCl-2 potentiometer, a 217-01 saturated calomel reference electrode (SCE), and a PCI-01 Cl^−^ indicator electrode, following ASTM D512-12 [30]. To ensure the results are representative and reliable, the Cl^−^ adsorption measurements were performed in duplicate for each formulation, and the average of the two values was adopted. The Cl^−^ adsorption capacities of LDO-CFA were calculated using Equation (1):

(1) $$Q_{3d}=35.5\;\times \;\frac{(C_{0}-C_{3d})V}{m}$$

where *Q*_3*d*_ refers to the 3-day Cl^−^ adsorption capacity (mg/g); *C*_0_ and *C*_3*d*_ represent the initial and 3-day Cl^−^ concentration (mM), respectively; *V* (L) denotes the volume of the solution; and m represents the mass of the adsorbent (g).

For the cement paste, the free Cl^−^ content of the paste was determined by Equation (2):

(2) $$Q_{e}=35.5\times \;\frac{\left(C-C_{0}\right)V}{m}\;\times \;100\%$$

where *Q_e_* denotes the free Cl^−^ content in the paste; *C* refers to the Cl^−^ concentration of the test solution (M); *C*_0_ represents the Cl^−^ concentration in deionized water (M); *V* stands for the solution volume (L); and *m* indicates the mass of the paste sample (g).

#### 2.5.2. Compressive Strength Test

The compressive strength of the cement paste specimen was measured using an electronic universal testing machine (CMT5504, Changchun Xinte Testing Machine Co., Ltd., Changchun, China). The test was conducted at a loading rate of 2 mm/min, with a load capacity range of 10 to 300 kN. The specimen dimensions were 20 mm × 20 mm × 20 mm. The average compressive strength was determined from six specimens, with outliers beyond ±15% of the mean excluded prior to the final calculation.

#### 2.5.3. Electrochemical Tests

Electrochemical measurements were performed on mortar specimens following 35 days of immersion in a 10 wt% NaCl solution using a VersaSTAT 3F potentiostat/galvanostat (Princeton Applied Research, New York, NY, USA). A conventional three-electrode configuration was employed for the electrochemical tests through open circuit potential (OCP) and electrochemical impedance spectroscopy (EIS) measurements, wherein the embedded steel rebar served as the working electrode, a stainless-steel plate as the counter electrode, and a LeiCi 217 saturated calomel electrode as the reference electrode. EIS was performed with a 10 mV perturbation (0.01 Hz–100 kHz) and fitted using Zsimpwin 3.60. Polarization curves were scanned at 0.5 mV/s. For electrochemical testing, a representative dataset was selected from the instrument measurements, and verification tests were performed for each mix formulation to ensure reliability.

### 2.6. Observation of Microstructure

The phase compositions of the synthesized LDHs-CFA, LDO-CFA, and cement hydration products were characterized by XRD (D8-Advance, Bruker, Bayern, Germany) using Cu Kα radiation. Using the KBr pellet technique, the Fourier-transform infrared (FTIR) spectra of specimens were collected on a Nicolet™ iS50 analyzer (Thermo Fisher Scientific, Waltham, MA, USA) for functional group identification. The specific surface areas of the LDHs-CFA and LDO-CFA were determined from N_2_ adsorption–desorption isotherms. The measurements were performed on a Micromeritics ASAP 2460 (Norcross, GA, USA), and the surface area was calculated using the Brunauer–Emmett–Teller (BET) method. The morphological characteristics were examined using TEM (Thermo Scientific Talos™ F200X, thermo Scientific, Brno, Czech Republic). The thermal behavior of the SPRC pastes was characterized by TG-DTG (1600HT, Mettler-Toledo, Stockholm, Sweden) from 30 to 1000 °C at 10 °C/min under argon. Using a Master-60 automatic mercury intrusion porosimeter (Micromeritics, Norcross, GA, USA) with a measurement precision of ±0.11%, the pore network of the hardened SPRC matrices was investigated.

## 3. Results and Discussion

### 3.1. Characterization of the Calcined LDO-CFA

**Figure 4:**
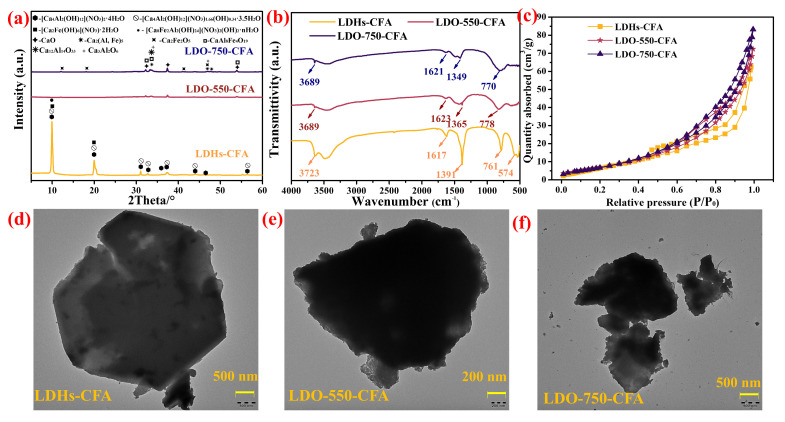
XRD patterns (**a**), FT-IR spectra (**b**), BET patterns (**c**), and TEM images of the LDHs-CFA, LDO-550-CFA, and LDO-750-CFA (**d**–**f**).

A comparison of the XRD patterns of LDHs-CFA, LDO-550-CFA, and LDO-750-CFA is presented in Figure 4a. Analysis of Figure 4a indicated that the main component was CaFeAl-NO_3_ LDHs, which can be referred to in the previous literature [29]. The materials displayed narrow, well-defined diffraction peaks at 2θ = 10.28°, indicative of high crystallinity and a regularly ordered layered arrangement. The presence of this characteristic peak near 2θ ≈ 10° in the LDHs’ diffractogram further verifies the successful incorporation of NO_3_^−^ ions into the interlayer spaces [17,31]. However, after calcination at 550 °C and 750 °C, the structure of LDO-CFA becomes partially degraded, resulting in a loss of crystallinity. Consequently, the XRD patterns no longer exhibit sharp peaks; instead, characteristic diffraction peaks corresponding to CaO, CaAl_8_Fe_4_O_19_, Ca_2_(Al, Fe)O_5_, Ca_2_Fe_2_O_5_, Ca_12_Al_14_O_33_ [31], and Ca_3_Al_2_O_6_ were observed (see Table 4). After calcination at 750 °C, LDO-750-CFA exhibited a lower XRD peak intensity compared to LDO-550-CFA and LDHs-CFA, accompanied by a more pronounced glass transition. This severe peak degradation suggests that the calcination process induces the loss of interlayer anions and structural water in LDHs, disrupts their crystalline structure, and triggers partial phase transformations [32].

FT-IR analysis was performed on the specimens to study the functional groups, and the results of LDHs-CFA, LDO-550-CFA, and LDO-750-CFA are presented in Figure 4b. As shown in Figure 4b, the LDHs-CFA specimen exhibited several characteristic absorption peaks. The absorption at 1617 cm^−1^ corresponded to the bending vibration of interlayer water (H–O–H). Peaks at 761 cm^−1^ and 574 cm^−1^ were linked to Al–OH stretching and Ca/Al parallel movement, respectively. Additionally, the peak at 1391 cm^−1^ was attributed to the asymmetric stretching of NO_3_^−^ [33]. However, for the calcined LDO-550-CFA and LDO-750-CFA, the absorption peaks associated with the Ca–OH stretching vibration near 3700 cm^−1^ and the H–O–H bending vibration at 1617 cm^−1^ were significantly weakened, while the NO_3_^−^ peak at 1391 cm^−1^ almost disappeared. These changes indicated a substantial loss of functional groups after calcination, consistent with the XRD results. Compared with LDO-550-CFA, LDO-750-CFA exhibited weaker and less distinct diffraction peaks, with some completely absent.

Figure 4c presents the nitrogen adsorption–desorption isotherms for LDHs-CFA, LDO-550-CFA, and LDO-750-CFA. The adsorption isotherms of LDHs-CFA, LDO-550-CFA, and LDO-750-CFA all correspond to Type IV, with specific surface areas of 28.99 m^2^/g, 37.55 m^2^/g, and 46.08 m^2^/g, respectively. Based on the BJH adsorption average pore diameters, the pore structures of these materials consist of both micropores and mesopores. The results also indicated that higher calcination temperatures lead to an increased specific surface area and total pore volume in LDO-CFA. This indicated that the calcination process induced dehydroxylation and the decomposition of interlayer anions, leading to a more porous and looser matrix in the LDO-CFA. This structural evolution resulted in a greater number of exposed active sites, thereby enhancing its Cl^−^ adsorption capacity.

These structural changes enhanced the material’s capacity to adsorb Cl^−^ in concrete solutions. The microscopic morphology of LDHs-CFA and its calcined derivatives (LDO-CFA) was examined using TEM (Figure 4d–f). As shown in Figure 4d, LDHs-CFA primarily exhibited a hexagonal flake-like structure, with an average diameter of approximately 3 μm. After calcination at 550 °C (Figure 4e), the crystal morphology of LDO-550-CFA remained flake-like but appears partially disrupted. When the calcination temperature was increased to 750 °C (Figure 4f), the morphology evolves into smaller flake-like grains, indicating noticeable refinement of the crystal structure.

**Figure 5:**
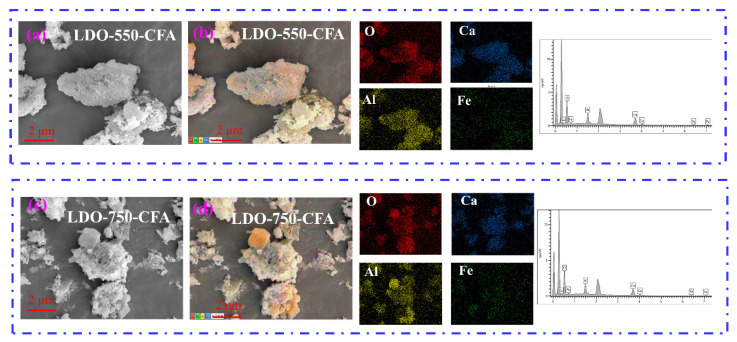
SEM images and mapping patterns of LDO-550-CFA (**a**,**b**) and LDO-750-CFA (**c**,**d**).

Figure 5 shows the SEM images and mapping patterns of LDO-550-CFA and LDO-750-CFA. Figure 5 reveals that calcination significantly altered the morphology of the LDHs, resulting in highly disordered structures for both the 550 °C and 750 °C derivatives. This effect was more pronounced in the LDO-750-CFA sample, which exhibited severe agglomeration of the layered sheets. Elemental mapping confirmed the uniform presence of Ca, Al, Fe, and O, affirming the formation of mixed-metal oxides after calcination.

### 3.2. Cl^−^ Adsorption Properties of Calcined LDO-CFA in CPSs

#### 3.2.1. Effect of Concentration on Ion Exchange Capacity

**Figure 6:**
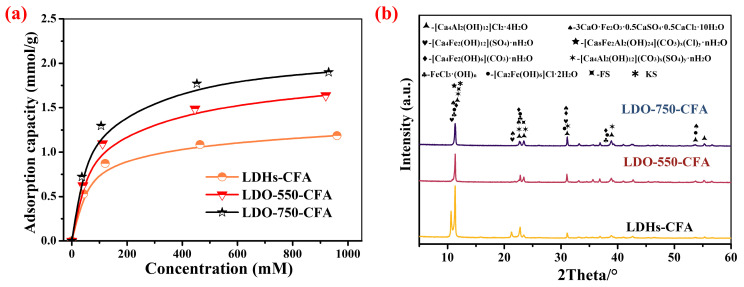
Cl^−^ adsorption performance of LDO-CFA in CPSs (**a**) and XRD patterns of the chloride-adsorbed products (**b**).

To evaluate the potential of the NO_3_^−^-intercalated hydrated talc for marine concrete applications, its Cl^−^ adsorption behavior was investigated directly in CPSs under multi-anion conditions. The Cl^−^ adsorption capacities were measured at initial Cl^−^ concentrations of 0, 60, 150, 500, and 1000 mM, as shown in Figure 6a. The experimental results indicated that the Cl^−^ adsorption capacity of LDO-CFA increased with higher calcination temperatures. This trend contrasts with previous studies [21,22], which suggested that LDHs lose interlayer anions between 400 and 600 °C and may undergo structural collapse at higher temperatures. However, this study showed that LDO-750-CFA, calcined at 750 °C, exhibited significantly enhanced Cl^−^ adsorption compared to both uncalcined LDHs-CFA and LDO-550-CFA. The XRD patterns in Figure 6b show that both LDO-550-CFA and LDO-750-CFA were reconstructed in the pore solution into LDHs-CFA, with Cl^−^ as the main interlayer anion, accompanied by minor amounts of CO_3_^2−^ and SO_4_^2−^. Notably, the peak intensity of LDO-750-CFA was significantly lower than that of LDO-550-CFA, indicating reduced crystallinity after reconstruction—a finding consistent with previous studies [32,34]. Additionally, a distinct shift in the main diffraction peak is observed after the LDO-CFA recovery process, with the 2θ angle moving notably from 10.28° to 11.34°. This shift confirms the successful reconstruction of the layered structure and an increase in the interlayer spacing, which is attributed to the replacement of intercalated NO_3_^−^ by larger CO_3_^2−^, SO_4_^2−^, and/or Cl^−^ from the solution. Furthermore, XRD analysis revealed that even trace amounts of competitor anions, specifically CO_3_^2−^ and SO_4_^2−^, could significantly impair the Cl^−^ adsorption capacity of the adsorbent. This inhibitory effect is primarily attributed to the stronger binding affinity (or higher adsorption priority) of CO_3_^2−^ and SO_4_^2−^ towards the LDO-CFA active sites compared to Cl^−^ [16,17,20,22,23]. In addition, the system formed specific compounds, such as KS, FS, and FeCl_3_(OH)_n_, which are absent in simulated solutions [16,20]. This disparity is attributed to the complex ionic composition of the actual concrete pore solution (containing Na^+^, K^+^, Ca^2+^, Cl^−^, SO_4_^2−^, CO_3_^2−^, etc.), which facilitates a wider range of chemical interactions and leads to the formation of the additional phase.

#### 3.2.2. Adsorption Isotherm Analysis

To elucidate the Cl^−^ adsorption mechanism, the experimental data were fitted with three adsorption isotherm models: Langmuir, Freundlich, and Tempkin [29,35]. According to the Langmuir isotherm theory, adsorption occurs as a monolayer on a surface composed of identical sites, each with an equal affinity for the adsorbate. It is commonly used to describe monolayer adsorption on homogeneous surfaces. The model, Equation (3), is expressed as follows:

(3) $$Q_{e}=\frac{bQ_{s}C_{e}}{1+bC_{e}}$$

where *Q_e_* represents the equilibrium adsorption capacity of Cl^−^ (mmol/g), *C_e_* denotes the equilibrium concentration of Cl^−^ in solution (mmol/L), *Q_s_* is the maximum adsorption capacity (mmol/g), and *b* is a constant related to the adsorption energy in the Langmuir model.

In contrast to the Langmuir model, the Freundlich isotherm assumes a heterogeneous surface facilitating multilayer adsorption with non-uniform site energies. Its slope (0 < 1/n < 1) serves as an indicator of the adsorption intensity and surface heterogeneity. The expression is Equation (4):

(4) $$Q_{e}=K_{F}C_{e}^{1/n}$$

Here, *C_e_* and *Q_e_* are the equilibrium concentration (mg/L) and adsorption capacity (mmol/g) of Cl^−^, respectively, and *K_F_* and *n* are constants correlated to temperature-dependent parameters.

The Tempkin isotherm incorporates adsorbent–adsorbate interactions and proposes a linear decrease in binding energy with increasing surface coverage. The model, Equation (5), is expressed as follows:

(5) $$Q_{e}=\frac{RT}{b}InK_{T}C_{e}$$

In these expressions, *b* is a constant related to the heat of adsorption (kJ/mol), and *K_T_* is the equilibrium constant indicative of the binding affinity. These values are estimated by linearly regressing the experimental data, where *Q_e_* is plotted against *C_e_*.

**Figure 7:**
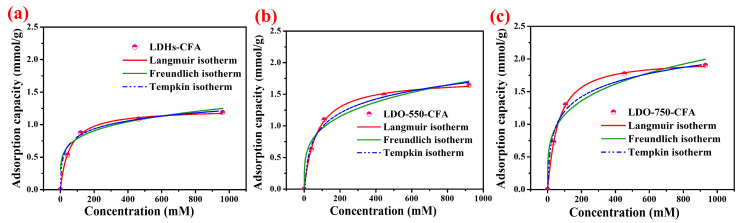
Chloride adsorption isotherms for LDHs-CFA (**a**), LDO-550-CFA (**b**), and LDO-750-CFA (**c**) fitted by the Langmuir, Freundlich, and Tempkin models.

The fitting results of the Langmuir, Freundlich, and Tempkin isotherm models are presented in Figure 7a–c, with the corresponding parameters summarized in Table 4. The suitability of each model was evaluated according to the correlation coefficient (R^2^). The Langmuir model yielded R^2^ values of 0.99 for all specimens, higher than those of the Freundlich and Tempkin models. This indicated that the Langmuir isotherm provides a better fit to the adsorption data, suggesting that the adsorption occurs via a monolayer mechanism on homogeneous sites. Thus, Cl^−^ adsorption by LDO-CFA in the CPSs can be described as single-molecule adsorption with equivalent and uniform active sites. As shown in Table 5, the LDO-750-CFA reached a capacity of 1.98 mmol/g in CPSs—60.1% and 13.6% higher than LDHs-CFA and LDO-550-CFA, respectively, which also exceeds the reported adsorbent in the literature by 41.47 mg/g (equivalent to ~1.17 mmol/g for Cl^−^) [16]. This result further confirms that the Cl^−^ adsorption capacity of LDO-CFA increased gradually with a higher calcination temperature. Furthermore, the achievable distribution coefficient (*K_d_*), calculated as the ratio of the adsorbed solute concentration to its equilibrium concentration in solution, was employed to quantify the adsorbent’s affinity for Cl^−^. The Cl^−^ adsorption affinity, quantified by *K_d_*, increased significantly with a higher calcination temperature. At a chloride concentration of 500 mM, the *K_d_* values were determined to be 81.89, 118.39, and 138.78 mL/g for LDHs-CFA, LDO-550-CFA, and LDO-750-CFA, respectively. This demonstrates a clear performance trend where the thermally treated LDO-750-CFA exhibits the strongest chloride affinity, with a *K_d_* value of 138.78 mL/g, significantly outperforming its counterparts. The data quantitatively confirms that a higher calcination temperature leads to a more effective adsorbent. Given the established consensus that CaFeAl-LDHs’ adsorption follows pseudo-second-order kinetics—a behavior shared by the calcined LDO—we focused our investigation on other aspects and did not reiterate the kinetic study [29].

### 3.3. Properties of Calcined LDO-CFA in Chloride-Containing Hardened SPRC Pastes

#### 3.3.1. Mechanical Properties

**Figure 8:**
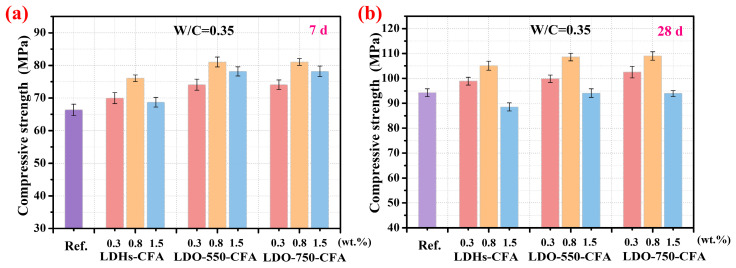
The effect of calcined LDO-CFA on the compressive strength of 7-day (**a**) and 28-day (**b**) SPRC pastes.

Figure 8 demonstrates the impact of calcined LDO-CFA on the development of compressive strength in the SPRC paste, measured at 7 days and 28 days. When the dosage of both LDHs-CFA and calcined LDO-CFA reached 0.8 wt.%, the compressive strength of the paste was significantly enhanced at both 7 days and 28 days. Notably, the LDO-750-CFA specimens achieved strengths of 81.05 MPa and 109.01 MPa at these ages, respectively. These values represented increases of 22.1% and 15.6% compared to the control one. Additionally, the observed reduction in compressive strength beyond the 0.8 wt.% threshold was due to the agglomeration of micro-nano materials. Excessive content hinders homogeneous dispersion, resulting in defective areas that compromise the mechanical integrity of the SPRC paste. The enhanced compressive strength of the calcined LDO-750-CFA SPRC paste was primarily attributed to two mechanisms. First, its high free water absorption capacity effectively lowers the initial water–cement ratio and provides internal curing. Second, the finer particles of calcined LDO-750-CFA participate in the hydration process, reducing porosity, increasing capillary pore tortuosity, and ultimately leading to a denser cement matrix [32].

#### 3.3.2. Chloride Ion Adsorption Properties

**Figure 9:**
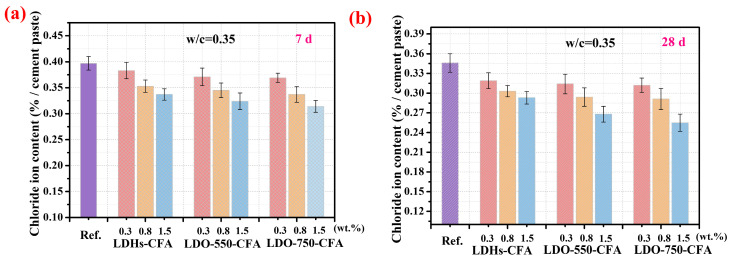
The effect of calcined LDO-CFA on the free Cl^−^ content of 7-day (**a**) and 28-day (**b**) SPRC pastes.

Figure 9a,b show the effect of LDO-CFA on the free Cl^−^ content in SPRC pastes at 7 days and 28 days, respectively. Experimental data reveal that with the addition of LDHs-CFA or LDO-CFA, the free Cl^−^ content in the SPRC paste gradually diminishes. At a LDHs/LDO content of 1.5 wt.%, the free Cl^−^ contents in the 7-day LDHs-CFA, LDO-550-CFA, and LDO-750-CFA specimens decreased to 0.337%, 0.324%, and 0.314%, respectively; this represents reductions of 15.1%, 18.4%, and 20.9% compared to the control one. Correspondingly, the free Cl^−^ contents in the 28-day paste specimens also reduced to 0.293%, 0.268%, and 0.255%. All the specimens demonstrated excellent Cl^−^ adsorption behavior. Among these three types of LDHs-CFA/LDO-CFA, the LDO-750-CFA-added cement paste exhibited the most pronounced Cl^−^ adsorption performance.

**Figure 10:**
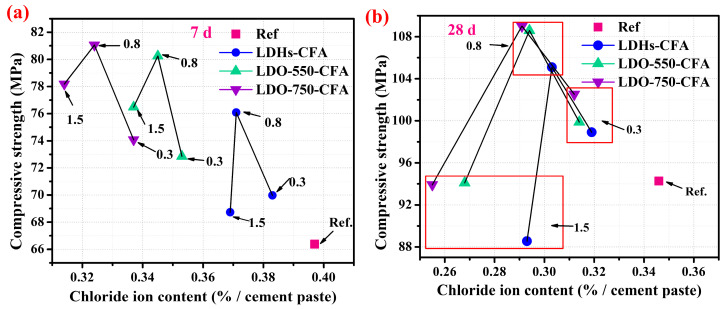
The quantitative relationships between compressive strength and free Cl^−^ content in the SPRC paste at 7 days (**a**) and 28 days (**b**), respectively.

Figure 10a,b show that both LDHs-CFA and the two calcined LDO-CFAs achieved an optimal balance between Cl^−^ adsorption and mechanical properties at a content of 0.8 wt.%. At this critical content, the LDHs-CFA, LDO-550-CFA, and LDO-750-CFA SPRC specimens achieved Cl^−^ adsorption efficiencies of 11.1%, 13.1%, and 15.1% at 7 days, respectively, while the compressive strength of the LDO-750-CFA-added paste reached 81.05 MPa. A similar trend was observed at 28 days, with the Cl^−^ adsorption increasing to 12.4%, 15.0%, and 16.1%, respectively, and the compressive strengths exceeding 100 MPa for all mixes. Notably, the LDO-750-CFA paste attained a strength of over 109 MPa. This study demonstrates a practical strategy of tailored LDO design for high-performance cement-based materials, which has enabled the development of a cement composite with a 28-day compressive strength higher than that reported in the current literature [36]. This synergistic effect stems from the dual functionality of LDHs: calcined LDO-CFA leverages its structural memory effect to regenerate the layered structure in an anionic environment, concurrently offering abundant Cl^−^ adsorption sites—a process enhanced by interlayer anion adsorption. Moreover, higher calcination temperatures for LDO-CFA increase the number of adsorption sites, and the resulting finer nanoparticles promote densification of the cement matrix. Consequently, in long-term assessments, the 0.8 wt.% LDO-750-CFA formulation demonstrates superior performance, exhibiting an outstanding Cl^−^ adsorption capacity while maintaining significantly higher compressive strength. This optimal combination of adsorption behavior and compressive strength makes it a promising candidate for applications requiring durable resistance against chloride-induced degradation.

### 3.4. Microstructure of Chloride-Containing LDO-CFA Cement Paste

#### 3.4.1. XRD

**Figure 11:**
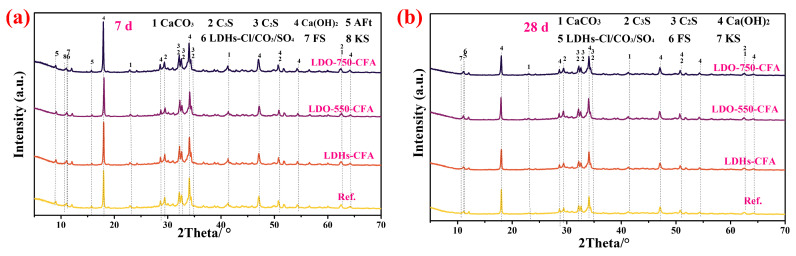
XRD analysis of 7-day (**a**) and 28-day (**b**) hardened LDO-CFA SRPC pastes with 3.5 wt.% NaCl.

XRD analysis of the hydration products was performed to examine the formation of LDHs-Cl. Figure 11 presents the XRD patterns of 7-day and 28-day SPRC pastes with NaCl and LDHs-CFA/calcined LDO-CFA. In Figure 11a, the Ca(OH)_2_ diffraction peak intensity was higher in pastes containing LDHs-CFA and calcined LDO-CFA than in the blank group, suggesting that both additives enhance early hydration and promote the formation of hydration products. Consequently, the peak intensities of C_2_S and C_3_S decrease. Moreover, calcined LDO-CFA was reconverted to LDHs-Cl in the NaCl-added system. Even LDO-750-CFA, calcined at 750 °C, effectively generates chloride-adsorbing LDHs-Cl. Figure 11b shows that the Ca(OH)_2_ diffraction peak intensities in cements with LDHs-CFA and calcined LDO-CFA were lower than in the control, suggesting these additives have limited influence on later hydration. Additionally, all systems contain some CaCO_3_ due to carbonation and the presence of CO_3_^2−^ and SO_4_^2−^ ions (Table 3). However, the CaCO_3_ content is slightly lower in pastes with LDHs-CFA and calcined LDO-CFA, likely because these materials adsorb CO_3_^2−^ and improve cement compactness, thereby reducing CO_2_ ingress from the air.

#### 3.4.2. TG-DTG

**Figure 12:**
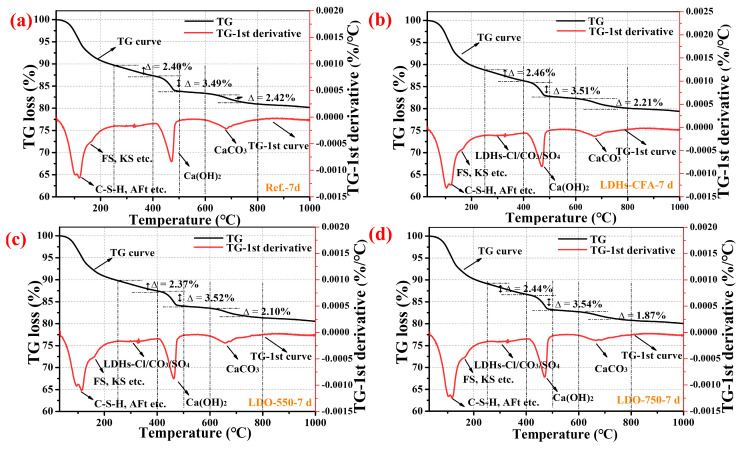
TG-DTG analysis of 7-day hardened SRPC paste with 3.5 wt.% NaCl: Ref. (**a**), LDHs-CFA (**b**), LDO-550-CFA (**c**), and LDO-750-CFA (**d**).

XRD analysis indicated that LDO-CFA primarily accelerates early-stage hydration with minimal impact on later phases; hence, subsequent microscopic analysis focused solely on the 7-day samples. Figure 12 presents the TG-DTG results of the 7-day cement pastes with NaCl and LDHs-CFA/LDO-CFA. As shown in Figure 12, the endothermic event at approximately 200 °C was associated with the loss of non-evaporable water due to the concomitant decomposition of several phases, namely C-S-H gel, KS, FS, and AFt, resulting from overlapping decompositions [37]. Between 250 and 400 °C, KS and FS partially decompose, losing six water molecules from the main layer. In pastes containing LDHs, some LDHs-CFA-Cl also decomposes within this temperature range. The endotherm at 400–500 °C corresponds to the decomposition of Ca(OH)_2_, while that at 600–800 °C is mainly due to CaCO_3_ decomposition [38]. Based on TG data, the Ca(OH)_2_ decomposition (observed between 400 and 500 °C) in the 7-day NaCl-mixed SPRC paste with LDO-750-CFA was 3.54%, exceeding that of the control. Additionally, the reduced CaCO_3_ peak intensity (600–800 °C) in LDO-750-CFA pastes suggests LDO-750-CFA-modified cement has higher compactness and better resistance to atmospheric CO_2_.

#### 3.4.3. Pore Structure

**Figure 13:**
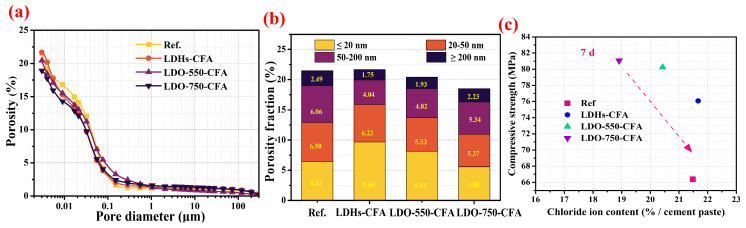
Total porosity (**a**), pore size distribution (**b**), and relationships between compressive strength and total porosity (**c**) of hardened SRPC pastes with 3.5 wt.% NaCl.

Figure 13a,b illustrate the pore structure characteristics of 7-day pastes with 3.5 wt.% NaCl through comparative analysis of cumulative pore volume evolution and pore size distribution patterns. Figure 13 shows the total porosity and pore size distribution of hardened SRPC paste with 3.5 wt.% NaCl and 0.8 wt.% LDHs/LDO. Figure 13a demonstrates that the LDO-550-CFA and LDO-750-CFA pastes exhibit a marginally lower total pore volume compared to the control one, with no statistically significant difference. The pore volume reduction observed in LDO-750-CFA-modified pastes (Figure 13b) primarily stems from decreased harmful pore counts across three critical ranges: 0.02–0.05 μm (gel pores), 0.05–0.2 μm (capillary pores), and >0.2 μm (macropores). Figure 13c illustrates the correlation between the compressive strength and total porosity for 7-day hardened SRPC pastes incorporating 3.5 wt.% NaCl. As widely recognized in cementitious systems, a definitive inverse relationship is observed, wherein higher porosity corresponds to diminished compressive strength [9,12]. This correlation primarily arises because pores act as intrinsic flaws or stress concentration points within the material matrix, effectively reducing its load-bearing capacity. Furthermore, beyond total porosity, specific pore structure characteristics—such as pore size distribution, connectivity, and shape—significantly influence mechanical performance. A refinement in these microstructural features, leading to a less pervious and more homogeneous matrix, could potentially enhance strength even at comparable overall porosity levels. As evident from Figure 13c, the total porosity and the proportion of harmful pores in the high-strength LDO-750-CFA sample are notably inferior to those in the other samples.

### 3.5. Corrosion Inhibition of LDO-CFA in Reinforced Mortars

#### 3.5.1. Open Circuit Potential

**Figure 14:**
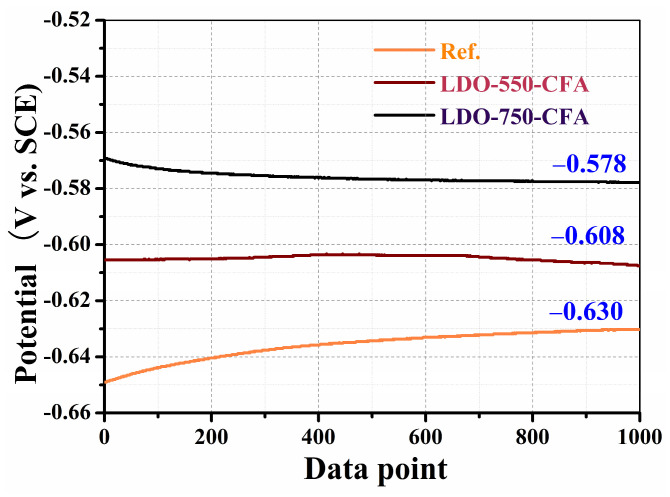
The OCP results for LDO-CFA specimens immersed in 10 wt.% NaCl solution over a 35-day period.

According to the corrosion mechanism of steel bars, the progression through the passivation, de-passivation, and active corrosion phases generates distinct electrochemical potentials at the interface between the steel and the surrounding environment [39]. Therefore, the OCP can be used as a qualitative indicator of the corrosion condition of steel bars in concrete. According to ASTM C876 [18,39], the corrosion probability of steel bars in concrete is assessed based on the OCP measured versus a SCE reference electrode. The criterion classifies the corrosion state into three conditions: an OCP more positive than −0.20 V indicates a passive state with a greater than 90% probability of no corrosion, but an OCP more negative than −0.35 V signifies active corrosion with a probability exceeding 90%. The OCP results for 0.8 wt.% LDO-CFA specimens immersed in 10 wt.% NaCl solution over the 35-day period are presented in Figure 14. As shown in Figure 14, the results indicated that following the 35-day immersion period, all specimens displayed corrosion potentials (relative to SCE) significantly lower than −0.35 V. These electrochemical readings, when evaluated against widely accepted corrosion standards, strongly suggest that the steel reinforcements had transitioned into an active state of corrosion. The progressive infiltration of Cl^−^ was the main cause of this result, as it triggered localized failure of the steel’s protective passive layer. The OCP values for specimens containing LDO-550-CFA (−0.608 V vs. SCE) and LDO-750-CFA (−0.578 V vs. SCE) were both higher than that of the control one (−0.630 V vs. SCE), with the most notable improvement observed in the LDO-750-CFA group. This suggests that LDO-750-CFA, due to its smaller particle size and higher Cl^−^ adsorption capacity, more effectively blocks Cl^−^ penetration and enhances the compactness of reinforced concrete.

#### 3.5.2. Electrochemical Impedance Spectroscopy Measurement

**Figure 15:**
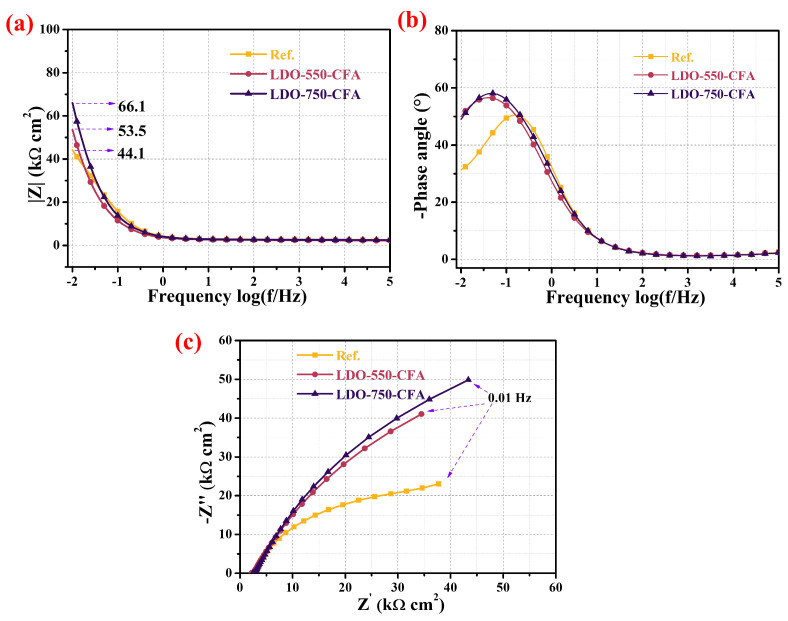
The EIS results for LDO-CFA specimens immersed in 10 wt.% NaCl solution over a 35-day period: Bode plots (**a**,**b**) and Nyquist plots (**c**).

EIS data is commonly represented through Nyquist plots and Bode plots, which facilitate the assessment of the corrosion resistance of steel bars by examining features such as the arc radius of the impedance spectrum, the impedance modulus, and the phase angle. A larger arc radius in the Nyquist plot, along with a higher impedance modulus in the low-frequency region and a more pronounced phase angle in the mid-frequency range of the Bode plot, generally corresponds to a reduced corrosion rate and the formation of a more stable protective film on the reinforcement surface [40]. Figure 15 presents the EIS results, displayed in both Nyquist and Bode plots, for the steel bar embedded in concrete incorporating 0.8 wt.% LDO-CFA. The study demonstrates a correlation between the low-frequency impedance modulus and the mid-frequency phase shift with respect to changes in the arc impedance. Research findings indicated that the low-frequency |Z|_0.01_ and mid-frequency phase angle are associated with alterations in the arc impedance. After the 35-day immersion period, the Nyquist plots in Figure 15a show that the low-frequency impedance (0.01 Hz) of the LDO-550-CFA and LDO-750-CFA specimens reached 53.5 and 66.1 kΩ·cm^2^, respectively—representing increases of 21.3% and 49.9% compared to the control one (44.1 kΩ·cm^2^). These results demonstrated enhanced corrosion resistance in the LDO-CFA composite, with the LDO-750-CFA specimen exhibiting the most significant improvement. Furthermore, the phase-angle Bode plots presented in Figure 15b reveal the presence of two time constants. Combined with an observed gradual decrease in the initial impedance modulus, this suggests a degradation in the protective properties of the mortar protective layer after the 35-day immersion period. The Nyquist plots presented in Figure 15c exhibit two distinct capacitive features: (1) a depressed semicircle in the high-frequency domain, characteristic of interfacial charge transfer kinetics at the mortar–steel junction, and (2) a low-frequency diffusion-limited tail approaching linearity, which correlates with elevated charge-transfer resistance at the electrochemical double layer and passive film formation on steel bars [41]. Notably, the LDHs-750-CFA-added mortar demonstrates a significantly larger semicircular arc radius compared to the control, suggesting an enhanced protective efficiency of LDO-750-CFA through optimized charge-transfer dynamics and improved barrier properties against steel corrosion. To summarize, at equivalent immersion durations, samples containing LDO-750-CFA exhibited a markedly greater arc radius and maximum phase angle relative to the Ref. group. These findings confirm that LDO-750-CFA effectively suppresses and delays the onset of rebar corrosion, underscoring its promise as a robust corrosion mitigation approach.

**Figure 16:**
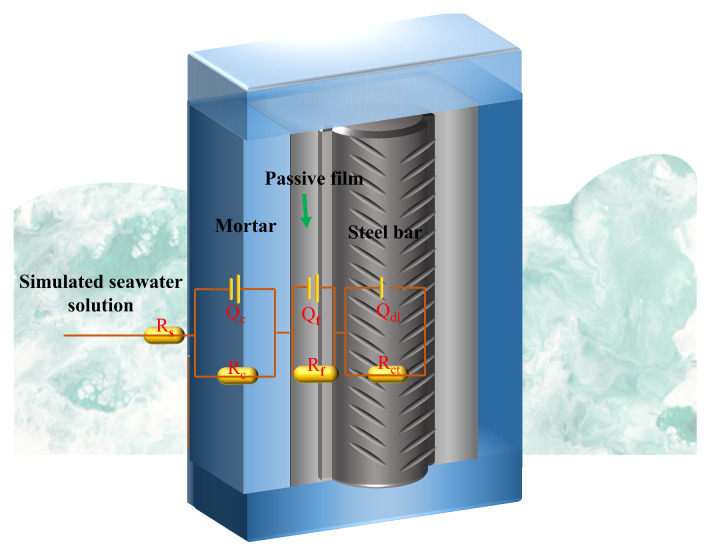
The equivalent circuit used to model the specimens within the reinforced mortar.

Quantitative assessment of corrosion components was conducted by fitting EIS data to the equivalent circuit model *R_s_*(*Q_c_R_c_*)(*Q_f_R_f_*)(*Q_dl_R_ct_*) (Figure 16). Here, the equivalent circuit elements are defined as follows: *R_s_* denotes the resistance of the simulated seawater solution. The time constant in the high-frequency region (*Q_c_R_c_*) corresponds to the mortar matrix resistivity and pore solution conductivity, where *R_c_* represents the pore solution resistance and *Q_c_* is the associated constant phase element. The mid-frequency range is described by R_f_ (passivation film resistance) and *Q_f_* (film capacitance). The low-frequency region (<10 Hz) is dominated by *R_ct_*, the charge transfer resistance, and *Q_dl_*, the double-layer capacitance at the steel surface [18,42]. The impedance is defined by Equation (6).

(6) $$Z_{CPE}=\frac{1}{Q}*{\left(j\omega \right)}^{-n}$$

where *Q* represents the CPE magnitude, ω is the angular frequency, and *n* is a dimensionless exponent between zero and one. The effective values for *C_f_* and *C_dl_* were subsequently calculated following the methods in Equations (7) and (8) [43]:

(7) $$C_{f}={\left(Q_{f}\right)}^{\frac{1}{n_{f}}}*{\left(\frac{1}{R_{f}}\right)}^{\frac{n_{f}-1}{n_{f}}}$$

(8) $$C_{dl}={\left(Q_{dl}\right)}^{\frac{1}{n_{dl}}}*{\left(\frac{1}{R_{dl}}+\frac{1}{R_{ct}}\right)}^{\frac{n_{dl}-1}{n_{dl}}}$$

The corrosion inhibition efficiency of LDO-CFA was evaluated based on electrochemical data using Equation (9) [39]:

(9) $$\eta \left(\%\right)=\;\frac{\left(R_{ct}-R_{0}\right)}{R_{ct}}$$

where *η* represents the corrosion inhibition efficiency, and *R_ct_* and *R*_0_ correspond to the charge transfer resistance of the LDO-CFA-added mortar and the reference group, respectively.

After 35 days of immersion in a 10 wt.% NaCl solution, both the LDO-550-CFA and LDO-750-CFA mortar specimens exhibited increased *R_c_*, *R_f_*, and *R_ct_* values compared to the control one. Specifically speaking, the *R_c_* value increased to 9.34 kΩ·cm^2^ for the control sample, and reached progressively higher values of 12.24 and 14.69 kΩ·cm^2^ for the LDO-550-CFA and LDO-750-CFA samples, respectively. This trend was consistent with the increases observed in the impedance arc radii and |Z|_0.01_ values, and collectively contributed to an enhancement in chloride resistance. Electrochemical analysis further demonstrated the superior performance of the LDO-750-CFA mortar, which exhibited significantly higher *R_f_* (7.21→10.96 kΩ·cm^2^, +34.2%) and *R_ct_* (63.8→92.4 kΩ·cm^2^, +44.8%) values, alongside lower *C_f_* (24.25→20.78 μF cm^−2^, −14.3%) and *C_dl_* (10.42→7.36 μF cm^−2^, −29.4%) values than the reference. The inhibition efficiencies of the LDO-550-CFA- and LDO-750-CFA-added mortars were 32.7% and 44.8%, respectively.

### 3.6. Discussion

**Figure 17:**
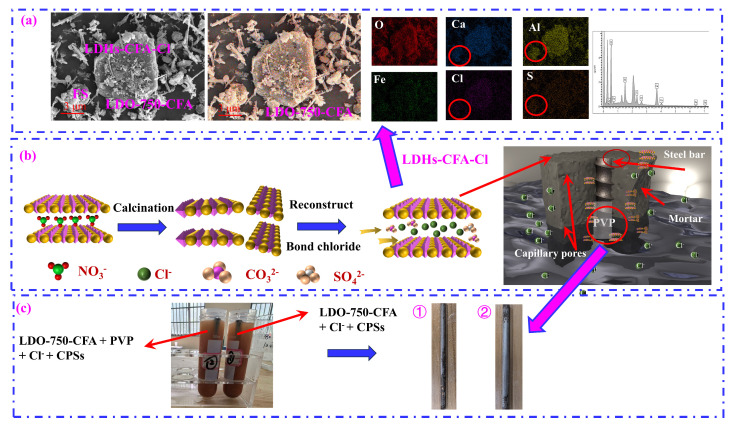
The schematic of the Cl^−^ adsorption and corrosion protection mechanisms for steel bars in mortar. (**a**) SEM-EDS mapping of the LDO-750-CFA sample for Cl^−^ adsorption from the CPSs; (**b**) Cl^−^ adsorption and corrosion protection mechanisms; (**c**) real pictures of PVP and LDO-750-CFA’s anti-corrosion effect in the simulated solution.

Building on these findings, this study explored how LDO-CFA synergistically enhances the Cl^−^ adsorption capacity within cement pore solutions, while also improving mechanical performance and corrosion resistance in SRPC composites. The Cl^−^ adsorption mechanism of the LDO-750-CFA specimens, as illustrated in Figure 17a,b, can be described as follows: First, LDO-750-CFA effectively captured Cl^−^ from the CPSs by leveraging its distinctive structural memory effect. The enhanced adsorption performance is attributed to the looser, more porous LDO-750-CFA matrix formed during calcination, which facilitated mass transfer and increased the active site density, consequently leading to superior adsorption. Second, the micro-nano-scaled two-dimensional LDO-750-CFA particles refined the pore structure of the cement paste through a micro-nano filling action [21,22]. This pore refinement decreased the overall porosity and increased the density of the cement matrix, which in turn limited the ingress of atmospheric CO_2_ and promoted greater Cl^−^ adsorption by LDO-750-CFA. The mechanical strength is attributed to a combination of LDO-750-CFA’s seeding, internal curing, and micro-filling effects [32]. Meanwhile, PVP promoted a homogeneous distribution of the LDO-750-CFA particles. This synergy refined the pore structure, creating a denser microstructure with lower porosity that enhanced the composite’s mechanical properties.

This anti-resistance improvement can be attributed to multiple synergistic mechanisms, specifically pore refinement and the increased tortuosity of the mortar, Cl^−^ adsorption by LDO-750-CFA, and the formation of a PVP-induced organic passive film on the metal surface, as well as the role of PVP in promoting the uniform dispersion of LDO-750-CFA within the mortar and preventing particle agglomeration, as shown in Figure 17b,c. In addition, the strong oxidizing power and hydrolysis precipitation ability of trivalent iron (Fe^3+^) in LDO-750-CFA enable the rapid formation of a dense protective passivation film on the reinforcing steel surface.

### 3.7. Practical Implications and Limitations

Future work on LDO-CFA includes, first, developing multifunctional composite systems with anti-corrosion and resistance to Cl^−^ penetration properties; second, optimizing synthesis parameters, such as the calcination temperature (400–1000 °C) and holding time, to enhance the material’s crystal structure, specific surface area, and structural memory effect for improved Cl^−^ adsorption and stability, alongside exploring LDO-CFA pre-treatment; and third, expanding applications beyond cement-based composites into areas like water treatment (ion adsorption) and functional coatings, while considering compatibility and integration with other systems. Given the energy demand of the current calcination process (750 °C, 3 h), future work will include an energy analysis and efforts to optimize the parameters, notably by shortening the holding time, to improve efficiency. Furthermore, the environmental benefits of LDO-CFA primarily stem from three areas: the utilization of waste resources, environmental remediation, and the provision of greener alternatives. For instance, by utilizing metal-laden industrial waste as raw materials and enabling the regeneration of spent adsorbents, LDO-CFA technology supports a circular economy that minimizes new resource intake and waste generation. Additionally,, the outstanding anion adsorption capacity of LDO-CFA makes it a highly promising material for environmental remediation. The primary environmental benefit of LDO-CFA in this study lies in its ability to drastically extend the service life of reinforced concrete by inhibiting steel corrosion, which in turn avoids the extensive resource use and emissions from future repair and rebuilding.

This study has certain limitations to be addressed in future work. Specifically, the calcination process requires a more detailed description, and the analysis of steel corrosion in mortar—including the corrosion area, products, and conditions—needs to be expanded. Relying solely on MIP fails to capture the actual 3D pore structure and connectivity, as it cannot distinguish between interconnected and isolated pores like BSE quantification or μ-CT can. This limitation thus leads to an incomplete understanding of the pore network. Additionally, this study also did not include a direct comparison with commercial inhibitors; such a benchmarking analysis will be the focus of future work to fully assess the practical application value of LDO-750-CFA.

## 4. Conclusions

1. The chemical composition and microstructure of LDO-CFA calcined at 550 and 750 °C were characterized by XRD, FTIR, BET, SEM mapping, and TEM. Calcination of the LDO-CFA system generates several crystalline phases, namely CaO, CaAl_8_Fe_4_O_19_, Ca_2_(Al, Fe)O_5_, Ca_2_Fe_2_O_5_, Ca_12_Al_14_O_33_, and Ca_3_Al_2_O_6_.
2. LDO-CFA shows a high Cl^−^ adsorption capacity in both CPSs and cement-based materials. The process follows the Langmuir model, suggesting uniform monolayer adsorption. LDO-750-CFA showed a peak capacity of 1.98 mmol/g in CPSs, surpassing LDHs-CFA by 60.1%. The long-term efficacy was confirmed in SRPC pastes, where free Cl^−^ was lowered to 0.255–0.293% after 28 days of curing.
3. At an optimal dosage of 0.8 wt.%, the LDO-750-CFA paste significantly improved the compressive strength of the SPRC paste, increasing it by 22.1% at 7 days and 15.6% at 28 days compared to the control. At 0.8 wt.%, the 28-day paste achieves an optimal balance, exhibiting 16.1% Cl^−^ adsorption efficiency while retaining a compressive strength exceeding 109 MPa.
4. Electrochemical tests revealed that, compared to the control, the LDO-750-CFA specimen improved the corrosion resistance of reinforced mortar by increasing the OCP, impedance arc radii, |Z|_0.01_ values, R_p_, R_f_, R_ct_, and inhibition efficiency, while reducing the C_f_ and C_dl_. It also delayed the initiation of steel corrosion.
5. This improvement originates from synergistic mechanisms within the mortar: LDO-750-CFA refines the pore structure and adsorbs Cl^−^, while the Fe^3+^ from it rapidly forms a protective passivation film. This effect is further boosted by PVP, which ensures the uniform dispersion of LDO-750-CFA and concurrently contributes an organic passive film.

By outlining a viable pathway for LDO-750-CFA composites, this study paves the way for more resilient and enhanced marine concrete structures.

## Figures and Tables

**Table 1:** Chemical composition of SRPC.

Elements	CaO	SiO_2_	Fe_2_O_3_	Al_2_O_3_	MgO	SO_3_	TiO_2_	K_2_O	Na_2_O	Others	LOI
Content (wt%)	58.93	18.88	4.41	4.00	3.99	3.40	0.62	0.51	0.24	1.97	3.05

**Table 2:** Ion concentrations of the main component in the CPSs of SPRC.

Components	Ca^2+^	Na^+^	K^+^	CO_3_^2−^	SO_4_^2−^	OH^−^
Content (mmol/L)	22.6	9.65	38.3	1.1	3.2	57.6

**Table 3:** Mix proportions of electrochemical experiment specimens (g).

Runs	SRPC	Sand	Water	LDO-CFA	PVP
Ref.	500	1000	200	0	0.1
LDO-550-CFA	500	1000	200	4.0	0.1
LDO-750-CFA	500	1000	200	4.0	0.1

**Table 4:** Phases identified in LDO-CFA, Cl^−^-adsorbed LDO-CFA, and cement paste with LDO-CFA.

LDO-CFA	Cl^−^-Adsorbed LDO-CFA	Cement Paste with LDO-CFA
CaO	[Ca_4_Al_2_(OH)_12_] Cl_2_·4H_2_O	CaCO_3_
CaAl_8_Fe_4_O_19_	[Ca_4_Fe_2_(OH)_12_] (SO_4_)_n_·nH_2_O	C_3_S
Ca_2_(Al, Fe)O_5_	[Ca_4_Fe_2_(OH)_12_] (CO_3_)_n_·nH_2_O	C_2_S
Ca_2_Fe_2_O_5_	[Ca_4_Al_2_(OH)_12_] (CO_3_)_x_·(SO_4_)_y_·nH_2_O	Ca(OH)_2_
Ca1_2_Al_14_O_33_	[Ca_8_Fe_2_Al_2_(OH)_24_] (CO_3_)_x_·Cl_y_·nH_2_O	3CaO·Al_2_O_3_·3CaSO_4_·32H_2_O (AFt)
Ca_3_Al_2_O_6_	CaO·Fe_2_O_3_·0.5CaSO_4_·0.5CaCl_2_·10H_2_O	LDHs-CFA-Cl/CO_3_/SO_4_
	CaO·Al_2_O_3_·CaCl_2_·10H_2_O (FS)	FS
	CaO·Al_2_O_3_·0.5CaCl_2_·0.5 CaSO_4_·10H_2_O (KS)	KS
	FeCl_3_·(OH)_m_	

**Table 5:** Parameters of adsorption kinetics models.

Adsorption Kinetics Models	LDHs-CFA	LDO-550-CFA	LDO-750-CFA
Langmuir isotherm model			
*b*	0.018	0.013	0.011
*Q_s_* (mmol/g)	1.24	1.75	1.98
R^2^	0.995	0.998	0.999
Freundlich isotherm model			
*K_F_*	4.877	3.849	4.000
*n*	0.305	0.290	0.361
R^2^	0.885	0.978	0.973
Tempkin isotherm model			
*b* (kJ/mol)	6.295	11.320	11.487
*K_T_*	0.971	0.217	0.405
R^2^	0.975	0.992	0.967

## Data Availability

The original contributions presented in this study are included in the article. Further inquiries can be directed to the corresponding authors.

## Nomenclature

SRPC	Sulphate-resistant Portland cement
LDHs	Layered double hydroxides
LDO	Layered double oxide
LDHs-CFA	CaFeAl-NO_3_-layered double hydroxides
LDO-CFA	CaFeAl-layered double oxide
CPSs	Concrete pore solutions
C_4_AF	Tetracalcium aluminoferrite
SCE	Saturated calomel reference electrode
EIS	Electrochemical impedance spectroscopy
FS	Friedel’s salt
KS	Kuzel’s salt
OCP	Open circuit potential
PVP	Polyvinylpyrrolidone
C_3_S	Tricalcium silicate
C_3_A	Ttricalcium aluminate
AFt	Ettringite
